# Metabolic Circuits in Sap Extracts Reflect the Effects of a Microbial Biostimulant on Maize Metabolism under Drought Conditions

**DOI:** 10.3390/plants11040510

**Published:** 2022-02-14

**Authors:** Kgalaletso Othibeng, Lerato Nephali, Akhona Myoli, Nombuso Buthelezi, Willem Jonker, Johan Huyser, Fidele Tugizimana

**Affiliations:** 1 Department of Biochemistry, University of Johannesburg, Auckland Park, Johannesburg 2006, South Africa; othibengkgalaletso3@gmail.com (K.O.); nephalipertunia20@gmail.com (L.N.); myoliakhona@gmail.com (A.M.); nombuson@uj.ac.za (N.B.); 2 International Research and Development Division, Omnia Group, Johannesburg 2021, South Africa; willem.jonker@omnia.co.za (W.J.); johan.huyser@omnia.co.za (J.H.)

**Keywords:** abiotic stress, biostimulant, drought, maize, network analysis, pathway analysis, PGPR, plant metabolomics, sap

## Abstract

The use of microbial biostimulants in the agricultural sector is increasingly gaining momentum and drawing scientific attention to decode the molecular interactions between the biostimulants and plants. Although these biostimulants have been shown to improve plant health and development, the underlying molecular phenomenology remains enigmatic. Thus, this study is a metabolomics work to unravel metabolic circuits in sap extracts from maize plants treated with a microbial biostimulant, under normal and drought conditions. The biostimulant, which was a consortium of different *Bacilli* strains, was applied at the planting stage, followed by drought stress application. The maize sap extracts were collected at 5 weeks after emergence, and the extracted metabolites were analyzed on liquid chromatography-mass spectrometry platforms. The acquired data were mined using chemometrics and bioinformatics tools. The results showed that under both well-watered and drought stress conditions, the application of the biostimulant led to differential changes in the profiles of amino acids, hormones, TCA intermediates, phenolics, steviol glycosides and oxylipins. These metabolic changes spanned several biological pathways and involved a high correlation of the biochemical as well as structural metabolic relationships that coordinate the maize metabolism. The hypothetical model, postulated from this study, describes metabolic events induced by the microbial biostimulant for growth promotion and enhanced defences. Such understanding of biostimulant-induced changes in maize sap pinpoints to the biochemistry and molecular mechanisms that govern the biostimulant–plant interactions, which contribute to ongoing efforts to generate actionable knowledge of the molecular and physiological mechanisms that define modes of action of biostimulants.

## 1. Introduction

The plant vascular system, composed of the xylem and phloem, serves as an interface between the environment and the protoplast. This extracellular (apoplastic) space is not only a barrier against stresses but also has multiple functions in metabolism and signal transduction [1]. The plant sap is a central conduit for the translocation and mobilisation of water and nutrients among plant organs, and a source-to-sink transportation system of metabolites [2,3]. Thus, the metabolic composition and circuits of the sap reflect the dynamics in the metabolism of the plant under specific physiological conditions. Studies have investigated plant defences against environmental stresses by analysing the metabolic landscape of the apoplast. Differential changes in the sap chemical space not only reflect the physiological state of the plant but also affect the growth and development of different parts of the plants [1,4]. Thus, unravelling metabolite profiles in the sap can provide valuable insights into biochemical and molecular events related to the plant health, growth and development or metabolic reprogramming underlying plant–biostimulant interactions [4].

Thus, reported herein is an elucidation of biostimulant-induced differential metabolic profiles in maize sap extracts, revealing thus metabolic pathways involved in this biostimulant–maize interaction. Plant biostimulants can be defined as microorganisms and/or substances which, when applied to plants, stimulate various plant natural processes to enhance the efficiency and uptake of nutrients, crop quality as well as plant tolerance to environmental stresses [5,6]. Microbial biostimulants, such as plant growth-promoting rhizobacteria (PGPR)-based formulations, have been shown to promote plant growth as well as enhance the plant resistance (induced systemic resistance) and immunity against both abiotic and biotic stresses [7,8]. However, mechanistic descriptions of these beneficial effects of PGPR strains or consortia on their hosts are still enigmatic. Furthermore, the lack of fundamental understanding of modes of action of biostimulants (at molecular and cellular levels) hampers the novel formulation of biostimulants and the implementation of these products into agronomic practices [9,10].

Hence, by applying metabolomics to decode metabolic circuits in maize plants treated with PGPR under normal and drought stress conditions, we hope to contribute to generating (actionable) knowledge in regards to metabolic events that define PGPR-induced morphophysiological effects in crop plants. Metabolomics, a multi-disciplinary omics science that focuses on metabolism, has positioned itself as a central pillar in systems biology and involves qualitative and quantitative analyses of low-molecular-weight compounds, namely metabolites (≤1500 Da) present in a biological system [11,12,13]. Interrogating plant metabolism through *omics* approaches, such as metabolomics, enables the decoding of the language of cells at a molecular level. In the context of biostimulant–plant interactions, such accurate metabolic models are imperatively essential for devising and designing a roadmap for the next generation of biostimulant formulations and strategies for high productivity and resilience to climate change [10,14].

## 2. Results and Discussion

Identifying and quantifying metabolic changes in the sap extracts composition can provide valuable insights into the long-distance signalling and metabolite translocation, pointing to the plant dynamic biochemistry and physiological processes [4]. As highlighted in the introduction, the study aimed at investigating the metabolic reconfigurations governing the effects of a microbial biostimulant on maize plants under both normal (well-watered) and drought greenhouse-controlled conditions. The microbial biostimulant used in this study was a consortium of five *Bacillus* strains (Section 3), referred hereafter as (microbial) biostimulant or simply PGPR. The study was designed to comprise four (4) treatment (T) groups: non-stressed with no PGPR application, non-stressed with PGPR application, drought stressed with no PGPR application and drought stressed with PGPR application. The maize sap extracts were analyzed on a liquid chromatography-mass spectrometry (LC-MS) system and different chemometrics methods and bioinformatics tools were applied to mine and interpret the spectral data (Section 3). The visual assessment of the resultant chromatographic profiles (Figure S1) obtained from the untargeted approach indicated differential changes in extracted metabolites, pointing to the alterations in the sap metabolite composition due to PGPR treatment.

**Figure 1:**
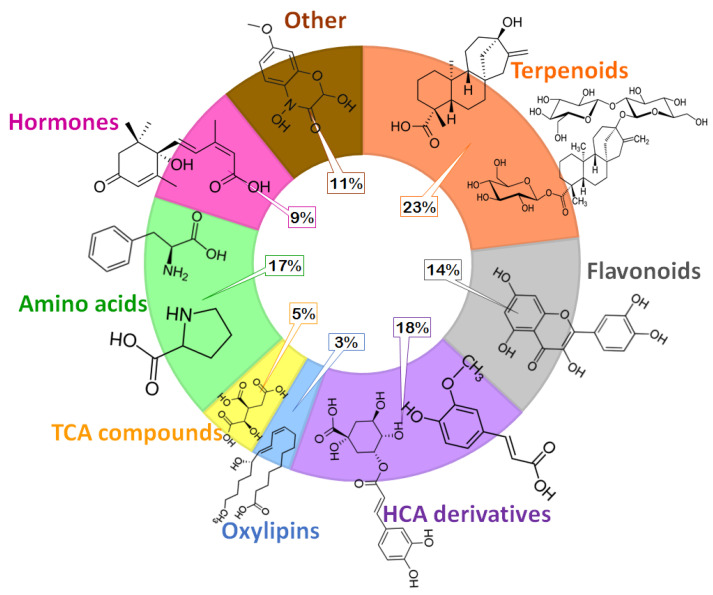
PGPR-induced metabolic changes spanned various classes of metabolites in sap extract. A doughnut chart showing the classification of all the putatively annotated metabolites from non-targeted analysis such as terpenoids, flavonoids, HCA derivatives, oxylipins and TCA compounds and amino acids, and hormones, which were identified using targeted analysis (Tables S1 and S2).

To further reveal the functional readouts of the cellular status of the maize metabolite transport/trafficking under various conditions, chemometrics methods were applied to mine LC-MS data. The exploration of the data by principal component analyses (PCA) models revealed treatment-related and time-related groupings (Figure S2A,B), suggesting differential metabolic profiles. Moreover, the annotation of these differentially abundant metabolites (Figure S2C) revealed that this metabolic landscape comprises classes such as organic acids, amino acids, hormones, lipids, flavonoids, hydroxycinnamic acid (HCA) derivatives, terpenoids and other compounds (Figure 1, Tables S1 and S2). To biologically articulate these mined differential changes in the extracted maize sap metabolome, two main aspects are pointed out, postulating thus a hypothetical framework that describes microbial biostimulant-induced reprogramming of maize metabolism towards (i) growth promotion and cellular priming, and (ii) a translation into enhanced resistance against abiotic stress, drought conditions in this case.

### 2.1. A Metabolome Map of Microbial Biostimulant-Induced Changes towards Growth Promotion 

**Figure 2:**
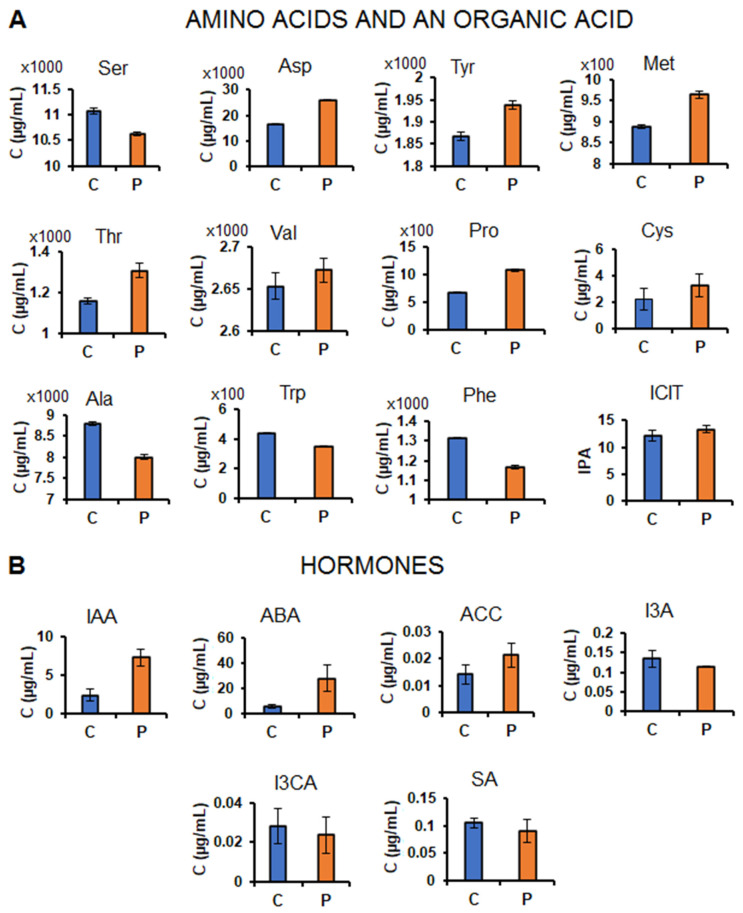
Effects of PGPR treatment on the levels of amino acids, organic acid and hormones in the maize sap. Bar graphs showing quantitative changes in the averaged concentrations (n = 9; i.e., 3 technical × 3 biological replicates) of amino acids and an organic acid (ICIT, isocitric acid) (**A**) and hormones (**B**) in the non-treated (control) versus the PGPR-treated groups, thus allowing for the investigation of the influence of PGPR treatment on plant growth and priming effect. For ICIT, the bar graph was generated using IPA (integrated peak area) instead of concentration level. Abbreviations: On the *x*-axis: C = control; P = PGPR-treated and on the *y*-axis: C = average concentration. The plotted error bars represent the standard deviation. For the full amino acid and hormone names, see Table S2. All the observed changes, except for Cys, I3A and I3CA, were statistically significant, and the respective *p*-values are also provided in Table S4.

The application of the microbial biostimulant induced differential changes on the amino acid profiles (Figure 2A). The levels of amino acids such as Met, Cys, Pro, Thr and Asp were increased in microbial biostimulant treated plants, whereas others such as Phe, Trp and Ser were found to be decreased and Val showed no change (Figure 2A). These observations resonate with a recent study by Rosier et al. [8], in which it was shown that inoculation with a PGPR strain, *Azospirillum lipoferum* CRT1 alters the maize xylem sap metabolite profiles including amino acids, carbohydrates and TCA cycle compounds, and these changes were associated with plant growth promotion. The high content of isocitric acid and some amino acids (Pro, Val, Met, Cys, Thr and Asp) found in the maize sap of PGPR-treated plants, under well-watered conditions (Figure 2A) suggests that the application of this microbial biostimulant leads to active transport of metabolites from the source/storage organ to a site where these metabolites are needed for various biological roles.

These increased amino acids and TCA compounds are involved in physiological processes associated with plant growth enhancement such as energy metabolism (TCA cycle and glycolysis), cell division, growth hormones regulation and protein biosynthesis [15,16,17]. For example, Thr is metabolized into Gly which plays an important role in plant photorespiration in the chloroplast (site for photosynthesis). Asp plays an important role in maintaining plant growth by serving as a substrate/precursor for the biosynthesis of four essential amino acids namely, Ile, Thr, Lys and Met via the Asp-family pathway [18,19]. Met is also involved in a wide range of functions in plant growth and development, i.e., it provides the required supply of sulfur and nitrogen to plants via its catabolism in the intracellular compartment, thus its presence in the sap extract (intercellular) (Figure 2A) suggests an enhanced mobilization of an important nutrient (nitrogen) between plant cells and organs. Moreover, Met is also known to maintain the structure of proteins required for cell differentiation and division [20,21]. It can also be transported to the roots to promote development of the latter by acting as a regulator of phytohormones such as auxins, brassinosteroids and cytokinin [22].

On the other hand, a decrease in the levels of other amino acids such as Ser, Phe and Trp observed in the sap of biostimulant-treated plants (Figure 2A) may suggest possible active utilization of these metabolites in the cellular milieu. For instance, these decreased amino acids could be used up in the process of protein synthesis, thus promoting plant growth and cell function [23,24]. Protein synthesis contributes significantly to plant growth by increasing biomass and/or producing proteins which play critical roles in metabolic pathways such as reaction catalysis, DNA replication, and transport of molecules inside and outside of the cells and serve as building blocks of cell membranes [25]. Furthermore, it can be postulated that the observed decreased levels of certain amino acids (in the sap of biostimulant-treated maize plants) relates to their active involvement in energy metabolism. For example, Ser and Trp can be degraded into pyruvate, while Phe is first converted to tyrosine which is then converted into fumarate in the mitochondria where energy is required to perform other growth-promoting mechanisms [26].

Additionally, the catabolism of amino acids also provides the carbon structural backbone required for the generation of a wide variety of defence-related secondary metabolites through the phenylpropanoid pathway [23,27]. For example, Trp serves as a precursor to compounds such as indoleamines, glucosinolates, alkaloids, phytoalexins and indole acetic acid (IAA), while Phe is a precursor to flavonoids, tannins, isoflavonoids, lignin, volatiles and anthocyanins [23,28]. Furthermore, a study done by Basha et al. [29], has shown that foliar treatment of chickpea with PGPR resulted in greater phenylalanine ammonia-lyase (PAL) activity as well as an accumulation of phenolic compounds as compared to the non-treated plants. Thus, the decreased levels of Trp and Phe in the sap (Figure 2A) may point to the possible activation of phenylpropanoid pathway in the cells, thereby suggesting the preconditioning of the plant to respond better to subsequent challenges. Moreover, the activation of the phenylpropanoid pathway forms part of the biostimulant-induced systemic response (ISR) in plants [30]. As mentioned above, Trp is also involved in the biosynthesis of auxins, which are responsible for regulating plant development and growth [31,32]. In this study, the content of IAA increased in the sap of biostimulant-treated maize plants (Figure 2B), reflecting active auxin transport which is positively correlated with plant growth promotion. This hormone enhances plant growth by increasing the length and surface area of the roots for enhanced nutrient and water uptake [33].

Other hormones which accumulated in the sap of maize plants treated with the microbial biostimulant (in comparison to non-treated plants), under well-watered conditions, included abscisic acid (ABA), 1-aminocyclopropane-1-carboxylic acid (ACC) and indole carboxylic acid (I3CA) (although the changes in I3CA was not statistically significant) (Figure 2B), which also points to their active transport via the sap. ABA coordinates a variety of processes in plants namely, primary root growth, seed dormancy and germination as well as the maturation of seeds [34]. ACC, on the other hand, is a precursor of ethylene, another phytohormone that plays an important role in promoting plant growth and development and has also been classified as a stress hormone [35,36]. Moreover, decreased levels of SA and indole carboxaldehyde (I3A) were found in the sap of biostimulants-treated vs. non-treated maize plants, although the observed decrease in I3A was not statistically significant (Figure 2B). The two indolic metabolites namely, I3CA and I3A, are known to be involved in the IAA biosynthesis; however, their exact biological roles are still unclear [37]. Thus, this partially elucidated metabolic landscape in maize plants treated with a microbial biostimulant (under normal conditions, Figure 2) describes the sap as a metabolically highly-active and dynamic milieu, revealing the effects of the microbial biostimulant on maize metabolism.

**Figure 3:**
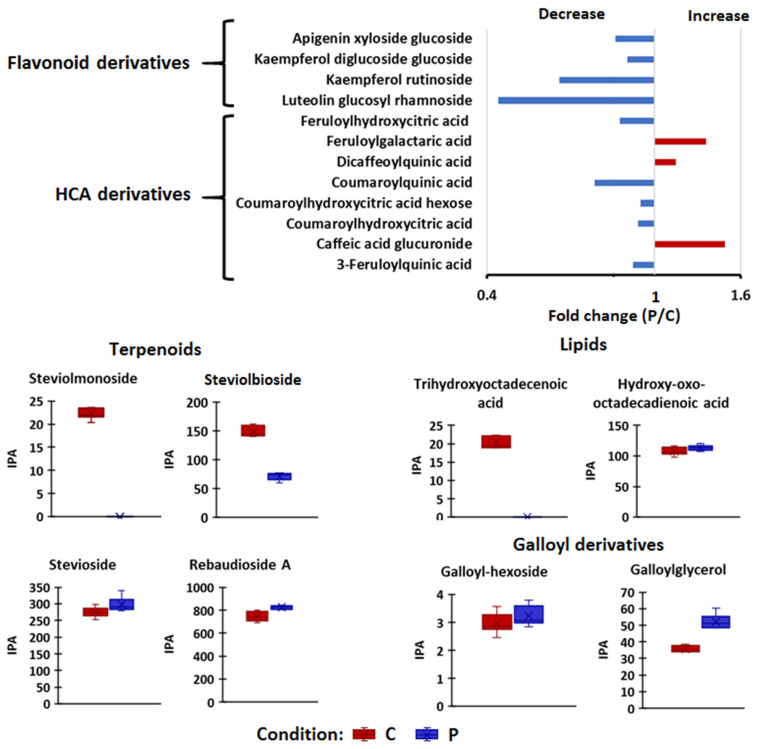
Effect of PGPR treatment on the secondary metabolites in maize sap under normal (non-stress) conditions. Relative quantitative profiles of selected metabolite classes namely, HCA- and flavonoid derivatives, terpenoids, lipids and galloyl-derivatives, in the sap of non-treated and PGPR-treated maize plants under normal conditions. The plotted error bars represent the standard deviation. Abbreviations: C = control, P = PGPR-treated, IPA = integrated peak area.

Furthermore, the biostimulant-induced reconfiguration of the maize metabolism, as reflected in the sap metabolic circuits, involved a differential alteration of secondary metabolites profiles, including flavonoids, hydroxycinnamic derivatives, terpenoids, lipids and galloyl derivatives (Figure 3). The levels of flavonoid glycosides such as apigenin xyloside glucoside, kaempferol diglucoside glucoside, kaempferol rutinoside and luteolin glucoside rhamnoside were decreased in the sap of biostimulant-treated plants compared to non-treated plants, under well-watered conditions (Figure 3). Flavonoids are synthesized in all parts of the plant and are involved in physiological processes such as gravitropism, signalling to symbiotic microorganisms and lateral root development [38,39,40]). Flavonoids also modulate auxin transport through direct and indirect interactions with cellular transport and regulatory mechanisms [41]. The levels of hydroxycinnamic acid derivatives such as feruloyl quinic acid, feruloyl hydroxycinnamic acid, caffeoyl hydroxycinnamic acid, coumaroyl hydroxycinnamic acid, coumaroyl hydroxycinnamic acid hexose and coumaroyl quinic acid were also decreased in the sap of PGPR-treated plants compared to untreated plants (Figure 3).

Terpenoids identified in this study are steviol glycosides (Figure 3). Rebaudioside D (3 isomers), rebaudioside I, rebaudioside F, rebaudioside A and stevioside were found in high levels in the sap of biostimulant-treated maize plants, under well-watered conditions (fold change > 1, *p*-value ≤ 0.1) whereas the levels of steviolmonoside and steviolbioside decreased due to the biostimulant application (Figure 3). Steviol can act as a precursor to C-13 hydroxylated gibberellins, growth-regulating phytohormones [42]. As such, the biostimulant-induced accumulation of glycosylated forms of steviol in the sap (Figure 3) can suggest that the application of this microbial biostimulant leads to active transport of these metabolites to other parts of the plants where the steviol glycosides are broken down into sugars and steviol, in which steviol is then converted to gibberellins. In support of our postulation, previous studies have shown that endogenous gibberellin content was found to significantly increase following the exogenous application of steviol [42]. Gibberellins are involved in the regulation of physiological processes such as leaf expansion, shoot growth, transition from vegetative to reproductive growth and fruit development. Translocation of gibberellins to other parts of the plant is crucial for plant growth and development; however, the long-distance movement of gibberellins seems to be restricted to its non-bioactive precursors (e.g., steviol) rather than to bioactive forms [43]. Furthermore, the glycosylation of compounds such as terpenoids is essential for functions related to accumulation, storage, and translocation within the plant [44].

Other classes of compounds that were measured are oxylipins and galloyl derivatives. Relative quantification levels of hydroxy-oxo-octadecadienoic acid showed no significant change in response to PGPR treatment, whereas the level of trihydroxyoctadecadienoic acid was decreased (Figure 3). Galloyl derivatives were higher in the sap of PGPR-treated plants (Figure 3). Taken together, the differential change in the amino acids, TCA metabolite, hormones, oxylipins, terpenoids and phenolics observed in this study under well-watered conditions, could point to a shift in the transport mechanisms leading to plant growth promotion and priming mediated by the applied microbial treatment. 

### 2.2. The Microbial Biostimulant-Induced Reprogramming of the Maize Sap Metabolome under Drought Conditions

**Figure 4:**
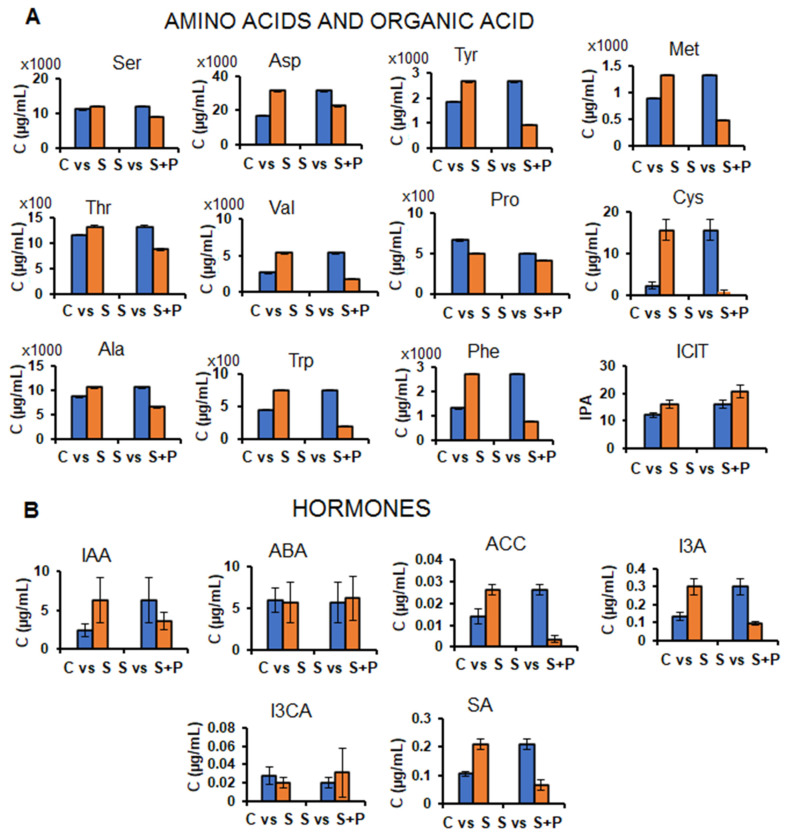
Effect of microbial-based biostimulant treatment on the maize plants under drought stress: Bar graphs showing quantitative changes in the average concentrations (n = 9; i.e., 3 technical × 3 biological replicates) of the selected amino acids and an organic acid (ICIT) (**A**) and hormones (**B**) (targeted analysis) in the maize sap of non-treated (control) versus the stressed groups and the stressed versus the PGPR-treated stressed groups, thus allowing for the investigation of the influence of PGPR treatment on plant response/tolerance to drought stress. For ICIT, the bar graph was generated using IPA instead of concentration as with the amino acids. The plotted error bars represent the standard deviation. Abbreviations: On the *x*-axis: C = control; S = stress; S+P = stress + PGPR and on the *y*-axis, C = average concentration; IPA = integrated peak area. For the full amino acid and hormone names, see Table S2.

The application of microbial-based biostimulant on maize plants under drought stress conditions resulted in differential alteration in the levels of amino acid, hormones, phenolics and lipids in the sap milieu. Quantitative changes in the inorganic ions and amino acids in the sap are thought to play a role in regulating osmotic potential of the phloem [4]. Under drought stress conditions, the levels of amino acid such as Met, Cys, Thr, Asp, Phe, Trp, Val and Ser were decreased in the sap of the biostimulant-treated maize plants compared to the non-treated plants (Figure 4A). Abiotic stresses such as drought can induce premature leaf senescence as a stress management strategy. Leaf senescence involves nutrient recycling and remobilization through physiological processes such as protein degradation [45]. During this process, the proteins in leaves are broken down, subsequently yielding free amino acids which are translocated through the phloem sap to other parts of the plant [46]. This could explain the increase of amino acids observed in the sap of the non-treated drought stressed plants (Figure 4). However, in the sap of biostimulant-treated plants (under drought conditions), low amino acids content was observed (Figure 4), suggesting that premature leaf senescence was not induced, pointing to the priming effects of the microbial biostimulant. Besides functioning as nutrient (nitrogen) reservoirs, amino acids are involved in other biochemical roles. For instance, aromatic amino acids (e.g., Trp and Phe) are involved in stomatal regulation, ROS scavenging and osmotic adjustment under stress conditions [47,48]. Phe particularly, has been suggested to play a role in the maintenance of the redox homeostasis of the phloem sap [4]. Moreover, Phe is a critical metabolic node that plays an essential role in the interconnection between primary and secondary metabolism in plants. The metabolism of Phe plays a central role in the channelling of carbon from photosynthesis to the biosynthesis of phenylpropanoids (defence-related metabolites) [49]. Moreover, Phe metabolism and Phe, Tyr and Trp biosynthesis are associated with efficient nitrogen recycling mechanisms [50,51].

Furthermore, under drought conditions, the levels of IAA, I3A, SA and ACC were found to decrease in the sap of biostimulant-treated maize plants whereas the level of ABA and I3CA showed no significant changes in the sap of biostimulant-treated plants compared to the non-treated plants under drought stress (Figure 4B). Chemical signalling under drought stress is mediated by the coordination of different hormones such as cytokinins, SA, ABA and IAA, amongst these ABA is known as the key hormone that accumulates in sap and is associated with the conservation of plant water availability via stomatal closure induction [52]. The function of IAA (and its derivative, I3A) in chemical signalling is yet to be elucidated [52]. Salicylic acid (SA) can also act as a signalling molecule under both biotic and abiotic stresses [53,54,55]. SA accumulation in the sap of non-treated stressed plants compared to control (Figure 4B), may suggest increased rate of SA transportation to facilitate the drought adaptation mechanisms (e.g., osmoregulation and antioxidation) in the cellular milieu [56]. However, with the application of PGPR, the level of SA in the sap was decreased (Figure 4B). This implies that PGPR priming of maize plants led to the rewiring of hormonal networks under drought stress conditions. Studies have shown that PGPR imparts drought tolerance via hormonal reprogramming [57,58].

**Figure 5:**
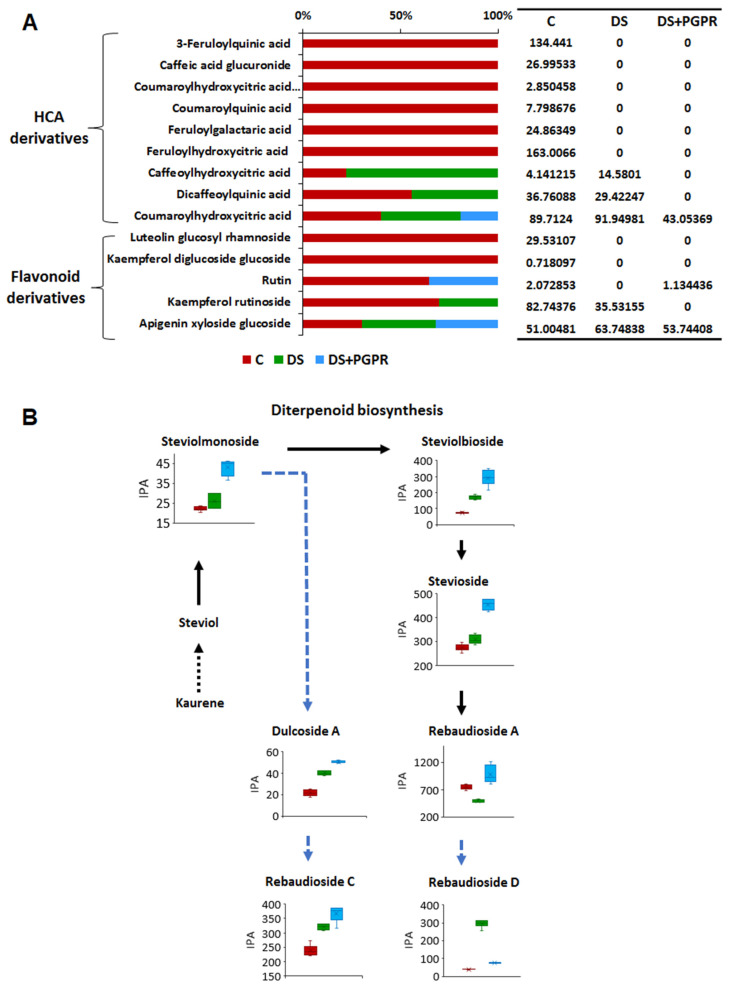
Relative quantification of phenolics and steviol glycosides. (**A**) Relative quantitative profile of HCA and flavonoid derivatives in the sap of non-treated (DS) and PGPR-treated (DS+PGPR) maize plants under drought stress conditions as compared to control (C, non-stressed and non-treated). The values represent the averaged (n = 6) of integrated peak area. (**B**) Pathway mapping of steviol glycosides. The plotted error bars represent the standard deviation.

Other metabolites that were measured in the maize sap include HCA derivatives and flavonoids and terpenoids. The application of microbial biostimulant on maize plants also induced differential alterations in the levels of these secondary metabolites in the sap extracts, under drought conditions (Figure 5). The relative levels of most of the secondary metabolites such as flavonoids and HCA derivatives were decreased (and others absent) in the sap extracts (from both non-treated and primed plants) (Figure 5A), suggesting their translocation into the active cellular milieu for different biological and defence-related functions. Several studies have observed increases in levels of phenolic compounds in drought stressed plants to combat the elevated levels of reactive oxygen species (ROS) induced by water-deficit stress or as a consequence of the reduced lignin biosynthesis in which the HCAs are precursors [59,60,61].

On the other hand, the levels of steviol glycosides (SG) such as steviolmonoside, steviolbioside, stevioside, rebaidioside A and C and dulcoside A were increased in the sap of biostimulant-treated plants compared to non-treated plants (Figure 5B). Under drought conditions, the SG content was increased in stevia leaves [42]. This increase in SG content could be a parameter for adapting to water stress conditions, thus maintaining high cellular integrity in plant tissues. This would allow the plant to maintain water at the cell level because of an increase in the intracellular osmotic potential [42,62].

### 2.3. Measured Changes in Sap Metabolic Profiles Reflect Alterations in Cellular Pathways 

**Figure 6:**
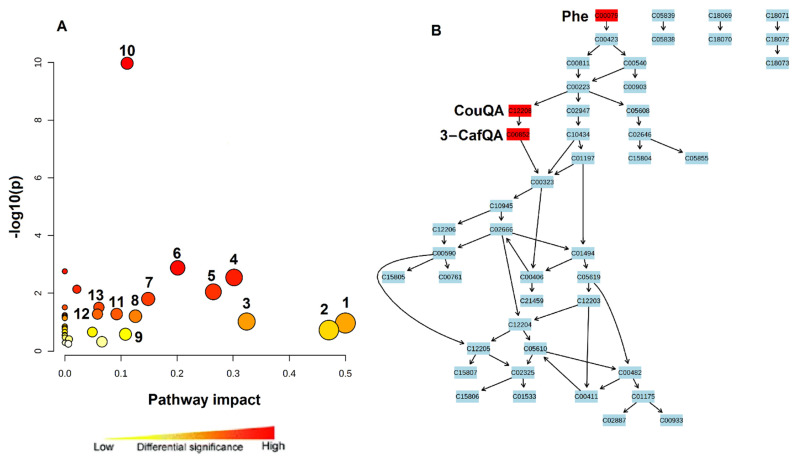
A typical synopsis of metabolic pathway analysis. (**A**) The graph displaying the ‘metabolome view’ containing all the mapped pathways arranged by *p*-values on the *y*-axis and the pathway impact (differential significance) on the *x*-axis. Each circle represents a metabolic pathway and the size of which is dependent on the impact of each pathway. Pathway impact values refer to the cumulative percentage from the matched metabolite nodes and the maximum importance of each pathway is 1. The different colours (from yellow to red) represent a gradation from low to high significance. (**B**) Topological characteristics as well as the phenylpropanoid biosynthesis pathway. The different colours indicate the differential significance of the match metabolites on a pathway. For the full names of the numbered pathways, see Table S3.

The measured metabolic circuits (in the maize sap), associated with microbial biostimulant-mediated drought stress tolerance in maize, implied a reconfiguration of maize metabolism, spanning a range of cellular pathways such as isoquinoline alkaloid biosynthesis, Phe metabolism, Trp metabolism and Gly, Ser and Thr metabolism as the most significantly altered pathways, amongst others (Figure 6; Table S3). The altered pathways span a broad range of biological roles including plant growth promotion, priming and drought stress alleviation. For example, Phe and Trp metabolism play key roles in channelling photosynthetic carbon to the biosynthesis of phenylpropanoids (defence-related metabolites), thus acting as the interconnection between primary (central carbon metabolism) and secondary metabolism in plants [27,49].

Another altered cellular pathway (as reflected by the decreased levels of Thr, Ser, and Asp in PGPR primed plants sap milieu under drought, Figure 4) is Gly, Ser and Thr metabolism (Figure 6; Table S3). The latter is involved in photorespiration; a biological process that is tightly linked to photosynthesis and is known to increase under drought conditions [63]. Correspondingly, Gly, Ser and Thr metabolism is one of the pathways that were significantly impacted in the leaves of PGPR-primed plants under drought [64,65]. Moreover, the changes in the sap milieu also reflected that Cys and Met metabolism is also altered intracellularly. This pathway is considered a central merging point of the three fundamental pathways of carbon, nitrogen, and sulfur assimilation, which are involved in plant growth, signalling and stress responses [66].

**Figure 7:**
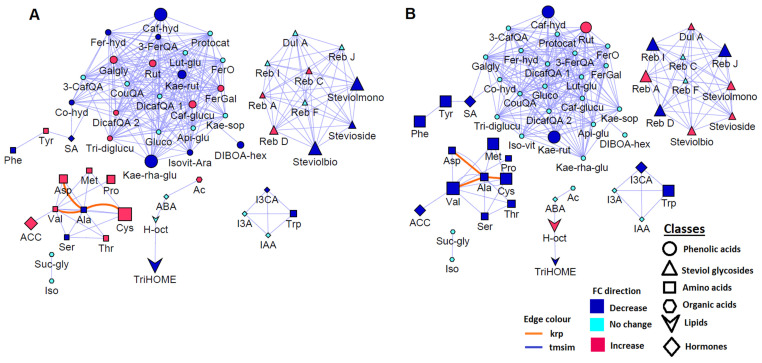
Metabolic network analysis: A biochemical and empirical network displaying metabolic relationship patterns between all the annotated metabolites in the sap milieu of PGPR-treated plants under (**A**) normal and (**B**) drought stress conditions. Metabolites are connected based on biochemical relationships (krp, Kegg reactant pairs) and structural similarity (tmsim, Tanimoto similarity). The direction of the arrow shows source-to-sink relationship. Each node represents a metabolite, the colour of the node shows the direction of the fold changes and the size of the node reflects the magnitude of the relative changes. The different shapes indicate the classes of the metabolites (see legend key on the bottom right).

Furthermore, correlation (metabolic) network analysis allowed the characterization of the complex relationship in measured metabolites (Figure 7). Correlation-based approaches build metabolite networks according to relational patterns observed in the experimental data and help identify altered graph neighbourhoods, which do not depend on any predefined biochemical pathways. Such mathematically constructed cartography allows the characterization of the molecular and cellular states induced by pathway interconnections under given experimental conditions. In the computed network, each metabolite is represented by a node, and in contrast to pathway analysis, the links between nodes correspond to the level of mathematical correlation between each pair of metabolites [67,68,69]. A biochemical/chemical similarity network analysis was accordingly applied to calculate and display relationship patterns between precursor and product metabolite reactant pairs, and molecules sharing a high degree of structural similarity, with Tanimoto coefficient ≥ 0.7 (Figure 7). 

Correlation network analysis revealed high interconnectivity of the metabolites (nodes) in the network independent of their biological pathways, demonstrating a high correlation of the biochemical (red) as well as structural (blue) metabolic relationships that coordinate the maize metabolism (Figure 7). Moreover, there were five (5) major clusters of various metabolite classes such as amino acids, phenolics, lipids, hormones and steviol glycosides, with phenolic acids making up the biggest cluster/hub, as well as a small cluster of succinylglycerol and isocitric acid (Figure 7). This observed differential clustering may point to possible varying regulatory events governing the PGPR-induced metabolic reprogramming in maize for plant growth promotion, priming and defence responses to drought stress. Furthermore, metabolic networking also allowed for the identification of significantly altered metabolites following the application of PGPR under both normal and stress conditions. For example, there was an observed increase in the levels of amino acids Met, Tyr, Pro, Asp, Val and Cys under normal, well-watered conditions (Figure 7A) as reflected in Figure 2A. This suggests that PGPR application induced an active transport of these amino acids to sites where they are needed for important biological roles, i.e., for physiological processes associated with plant growth promotion such as protein biosynthesis and growth hormone regulation. Conversely, Trp and Phe levels decreased pointing to their roles as precursors for key metabolites. That is, Trp serves as a precursor for growth promoting indole acetate hormones as shown in the I3CA, I3A and IAA cluster while Phe is a precursor for the biosynthesis of defence-related metabolites, i.e., flavonoids and HCA derivatives, suggesting possible priming of maize plants for subsequent challenges (Figure 7A).

Moreover, following application of PGPR under drought stress conditions, all amino acids decreased in levels possibly pointing to their roles as nitrogen reservoirs as well as in ROS scavenging and stomatal regulation, particularly the aromatic amino acids Phe and Trp, under stress conditions (Figure 7B). Furthermore, paying attention to the phenolics cluster, only kaempferol rutinoside, rutin and caffeoylhydroxycitric acid were significantly altered as opposed to the other phenolic acids which showed no significant changes following PGPR application in drought stress conditions (Figure 7B). This may suggest that one of the stress alleviation mechanisms induced by PGPR involves the upregulation/downregulation of specific phenolic compounds only. Furthermore, comparing the effect of PGPR application on the metabolism of maize plants under normal vs. stress conditions, it was observed that an increase in some metabolites (i.e., most amino acids and steviol glycosides) was induced under normal conditions while the opposite was observed under stress conditions. This points to the differential active transport of specific/certain metabolites from source to sink and vice versa) for various biological roles such as growth promotion under normal conditions and stress alleviation under drought stress conditions.

**Figure 8:**
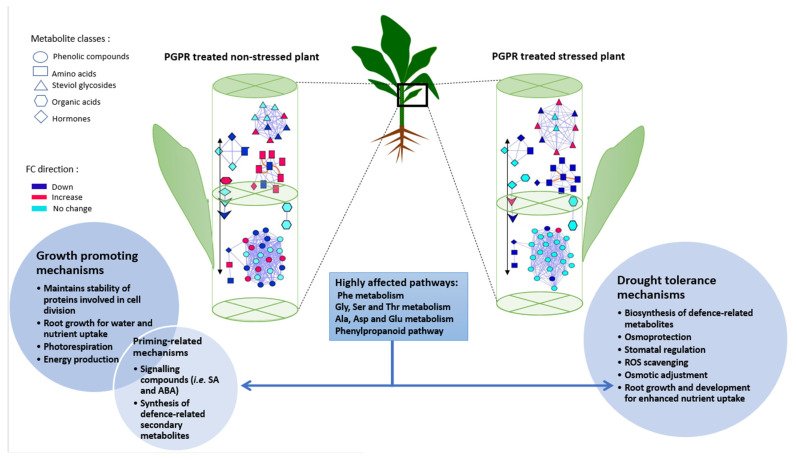
Some of the general metabolic and physiological events in a plant responding to PGPR treatment under normal and drought stress conditions: A contextual summary of the metabolic and biochemical events in PGPR-treated maize plants under normal (left) and drought stress (right) conditions which are translated into a variety of mechanisms through which plant treatment with PGPR induces priming effects, alleviates abiotic stresses (drought) and enhances plant growth under the respective conditions.

The reconfiguration of the maize metabolism in response to PGPR treatment under normal (well-watered) and drought stress conditions is reflected by differential changes in organic acids, phenolic compounds, steviol glycosides and amino acid levels in the sap. Thus, emerging from our results is a hypothetical model that describes metabolic events induced by the microbial biostimulant for growth promotion and enhanced defences (Figure 8). Biochemical and physiological events, induced by the microbial biostimulant in maize plants towards growth promotion include (i) enhanced photorespiration, (ii) improved energy production, (iii) improved root growth for enhanced nutrient and water uptake and (iv) maintained stability of proteins involved in cell division, as well as (v) priming-related mechanisms such as the synthesis of defence-related secondary metabolites and signalling compounds (Figure 8). On the other hand, the drought tolerance mechanisms induced by PGPR application under stress conditions include (i) induced biosynthesis of defence-related metabolites, (ii) enhanced ROS scavenging, (iii) improved osmoprotection, osmotic adjustment and (v) improved root growth and development for enhanced nutrient uptake (Figure 8). This postulated model of microbial biostimulant-induced mechanisms under normal (well-watered) and drought stress conditions complimentarily correlates to the framework suggested by Nephali et al. [65], in which the key mechanisms mediated by PGPR in drought tolerance were found to be, (i) enhanced stomatal conductance, antioxidant machinery and osmoregulation, (ii) enhanced cell wall formation. (iii) energy production, and (iii) remodelling of the plasma membrane.

## 3. Materials and Methods

### 3.1. Plant Cultivation and Treatments

BACSTIM 100 (Omnia Group Ltd., Bryanston, South Africa) was the microbial formulation that was used in this experiment. BACSTIM 100′s composition: consortium of five *Bacili* strains (viable PGPR) in the form of two strains of *Bacillus licheniformis*, two strains of *Brevibacillus leterosporus* and one strain of *Bacillus amyloliquefaciens*. The biostimulant formulation used produces spores and is commercially tested for stability (Omnia Group Ltd., Bryanston, South Africa). BACSTIM 100 as a commercial biostimulant product is interchangeably referred to as PGPR, biostimulant or microbial biostimulant in the current study. The experiment variables consist of an experimental control group with no stress and no PGPR treatment, and treated groups: P/PGPR (no stress + PGPR treatment), DS (drought stress + no PGPR treatment) and DS + PGPR (drought stress + PGPR treatment). Three biological control pots containing 5 plants per treatment were harvested at each time point.

PAN 3Q-240 maize (*Zea mays*) plants were cultivated in 17g of sandy soil. Sandy soil (pH 4.6) with a composition of 0.22% organic carbon, 1495 kg·m^−3^ bulk density and 0.38% m/m organic matter. Plants were placed on a rotating table and were grown in a greenhouse at Omnia facilities in Sasolburg, Free State, South Africa. In each pot, a fertilizer band containing 30 kg N/ha, 30 kg P/ha and 30 kg K/ha was placed in the middle and five maize seeds were placed in a row on either side of the band, 5cm apart and 3cm in depth. Immediately after the emergence of plants, thinning was applied and 5 healthy and uniform plants were chosen in each pot. Three weeks after the emergence (WAE) (3 leaf stage), all plants were top-dressed with 30 kg N/ha. PGPR treatment was applied at a rate of 2 L/ha during planting in soil furrow containing seeds. To promote seed germination, all plants were irrigated with 90% plant available water (PAW). At the 3-leaf stage, drought stress was applied: water levels were dropped 50% PAW and were maintained at 90% PAW for the non-stressed plants throughout the experiment. The equations contained in Supplementary File (Section S2.1) were used in calculating the 50% and 90% PAW, respectively.

### 3.2. Metabolite Extraction

Plant leaves were harvested one week after drought conditions were applied and this was done at 5 WAE for all biological replicates and treatments. During harvesting, the plant leaves and stems were cut off followed by rapid plunging of these plant materials in liquid nitrogen to quench enzymatic activity. Collection of the plant sap, a fluid that consists of a wide range of metabolites transported via the xylem and phloem from source to sink organs, was done using a high-pressure extraction method. Briefly, the sap from the plant material is extracted with custom build high-pressure stainless-steel presses (Omnia Group, Ltd., Johannesburg, South Africa). These presses can apply pressure up to 60 bar per cylinder, to maximise sap extraction. The collected sap extracts were then dried to completeness using a vacuum concentrator (Eppendorf, Merck, Johannesburg, South Africa). LC-MS grade methanol (50%) in a volume of 500 µLwas used to re-suspend the dried material after which, the re-suspended plant material was filtered into UPLC glass vials (Shimadzu, South Africa) and quality control (QC) samples were collected from all samples. 

### 3.3. Data Acquisition using Ultra-High-Performance Liquid Chromatography-High Definition Mass Spectrophotometry (UHPLC-HDMS) for Untargeted Metabolomics

UHPLC-MS was used in analysing the extracts. An Acquity UHPLC system (Waters Corporation, Milford, CT, USA) was used for the chromatography and a conditioned autosampler set at 4 °C was utilized. For the chromatographic separation, 2 µL of sample was injected into the UHPLC system (reverse phase Water Analytical C18 column, HSS T3 (1.8 µm, 2.1 × 150 mm) thermostatted at 60 °C). At a constant rate of 0.4 //mLmLmLmin, the metabolites were eluted through a gradient elution system using a degassed binary solvent: solvent A (0.1% aqueous formic acid) and solvent B (0.1% formic acid in acetonitrile. The starting conditions of 98% solvent A and 2% solvent B were held for 1 min. Gradually, the conditions were changed to 30% solvent A and 70% solvent B at 14 min and at 15 min they were changed to 5% solvent A and 95% solvent B. The changes were held for 2 min and changed back to the starting conditions at 18 min. Calibration of the analytical column was allowed for a period of 2 min before the next injection. The chromatographic run took 20 min in total.

For the MS analysis, the chromatography system was interfaced in-line with a SYNAPT G1 Q-TOF mass spectrometer (Waters Corporation, Milford, MA, USA) equipped with an electrospray ionization (ESI) source. V-optic mode was selected for the operation of the time-of-flight (TOF) analyser. A scan range of 50–1200 Da, scan time of 0.1 and an inter-scan delay of 0.02 s were the parameters used in acquiring the centroid data in both ESI positive and negative modes. The MassLynx^TM^ software (Waters Corporation, Machester, UK) enabled the automatic correction of the centroid mass values using the reference calibrant (leucine encephalin) in the samples for small deviations from the exact mass measurements, giving mass accuracies of between 1 and 3 mDa. The conditions for the MS instrument were set as follows: source temperature at 450 °C, sampling cone at 30 V, capillary voltage of 2.5 kV, extraction cone at 4 V, cone gas flow at 50 L/h, desolvation gas flow at 550 L/h. MS^E^ (a data-independent (DIA) method) was applied as follows: MS analysis was set to simultaneously perform non-fragmented and five fragmented experiments by applying an alternating collision energy of 0 eV (unfragmented) and from 10–50 eV (fragmented). MS^E^ was applied in order to produce molecular fragment information for downstream applications such as structure elucidation and compound identification. MassLynx 4.1 (SCN 704, Waters Corporation, Milford, MA, USA) was the software used to perform data manipulation and to control the hyphenated system. QC samples were used in order to assess the reliability, reproducibility and for non-linear signal correction. The background noise was monitored using blank samples (50% aqueous methanol) which were run randomly.

### 3.4. Data Acquisition on the LC-ESI-QqQ-MS System for Targeted Metabolomics

The standards used in this study were amino acid and phytohormone standards with a purity of ≥98% and were obtained from: Sigma (Johannesburg, South Africa), BDH (Johannesburg, South Africa) and Merck (Johannesburg, South Africa). The preparation of mixed working solutions of amino acids and phytohormones, with a concentration range of 25–1000 µg/L, was done using 50% aqueous methanol (Romil, Cambridge, UK) and solutions were stored at 4 °C. The LCMS-8050 (Shimadzu, Kyoto, Japan), a triple quadrupole mass spectrometry platform equipped with an ESI source and ultra-fast liquid chromatography (UFLC) as a front-end, was used. To quantify (absolute quantification) the targeted metabolites, the reaction monitoring (MRM) method was used. Direct infusion (using ESI source of MS) was used for the development and optimization of the MRM-MS. An integral component of the LabSolutions LCMS software (Shimadzu Corporation), the “MRM optimization method tool”, was used in the optimization of the collision energy (CE) for each transition. The tool automates the optimization of CE by collecting product ion scan data and by finding optimum CE for each transition. Table S2 contains all the reported MRM optimal conditions.

The UFLC system was fitted with Shim-pack GIST C18 column (2 µm, 100 × 2.1 mm I.D) (Shimadzu, Kyoto, Japan) and was used to analyse both the samples and the working solutions of the standards at 40 °C. Gradient elution at a constant flow rate of 0.2 mL/min was done for the chromatographic separation using solvent A (MilliQ water with 0.1% formic acid) and solvent B (methanol with 0.1% formic acid) (Romil, Cambridge, UK). The gradient elution of the amino acid standards was performed as follows: Starting conditions of 98% solvent A and 2% of solvent B held for 1 min. Conditions were changed from 1 to 3 min to 95% solvent A and 5% solvent B and at 3 min the conditions were changed to 90% solvent A and 10% solvent B held for 2 min. After the 2 min, the conditions were changed to 50% solvent A and 50% solvent and held for 2 min followed by a change back to the starting conditions. The chromatography run took 10 min and 3 µL was the injection volume. For the hormone analysis, the gradient elution was as follows: 98% solvent A and 2% solvent B over 0–3 min, 90% solvent A and 10% solvent B over 3–6 min, 80% solvent A and 20% solvent B held over 6–30 min and 95% solvent A and 5% solvent B over 30–38 min, 85% solvent A and 2% solvent B over 38–40 min. The chromatographic run took 40 min and 1 µL was the injection volume. Table S2 contains all the optimal conditions for MRM-MS analysis. The MS instrument conditions were as follows: nitrogen gas used as a drying gas at 10 L/min flow rate and as a nebulization gas at 3 L/min flow rate, heating gas at 10 L/min, interface temperature at 300 °C, interface voltage at 4 kV Dl temperature at 250 °C and heat block temperature at 400 °C.

### 3.5. Data mining: Data Processing and Multivariate Data Exploration

The centroid ESI positive and negative data generated from UHPLC-HDMS analysis were both analysed. MassLynx XS^TM^ 4.1software (Waters Corporation, Manchester, UK) was used for data processing and visualization. Data pre-processing was done using the MarkerLynx application manager provided by the MassLynx XS^TM^ software. MarkerLynx was also used for producing a data matrix of retention time (Rt)-*m*/*z* variable pairs with *m*/*z* peak intensity for each sample. For both ESI positive and negative data, the following Markerlynx parameters were set: to analyse the 0.8–15 min Rt range of mass chromatograms, *m*/*z* domain of a mass range of 50–1200 Da, mass tolerance of 0.05 Da and intensity threshold of 100 counts. The Rt was allowed to differ by ±0.2 min and ±0.05 Da for *m*/*z* values. This was performed for the alignment of peaks across the samples. Total ion intensities of each defined peak together with intensities prior to computing were used for normalisation. The processed and now cleaned data matrix was then exported to SIMCA (Soft independent modelling of class analogy) software, version 15 (Umetrics, Umea, Sweden) for data mining. In this study, unsupervised principal component analysis (PCA) was used for the exploration of the data. Further statistical analysis was done specially for the targeted analysis using MetaboAnalyst v 4.01 which included PLS-DA and the generation of heatmaps for the quantitative description of the results.

### 3.6. Metabolite Annotation and Biological Interpretation

The metabolites were annotated using a multistep workflow which corresponded to level 2 as classified by the Metabolomics Standard Initiative [70]. In this study the metabolites were annotated as follows: (i) molecular formula (MF) derived from full-scan accurate mass data (chemical and heuristic rules incorporated in MarkerLynx formula generator algorithm) in the form of mass differences, nitrogen rules, restrictions of element numbers, isotopic fit and ring-and-double-bond equivalent [71,72]; (ii) Dictionary of Natural Products (DNP), Chemspider and PlantCyc were the databases and bioinformatics used for searching the selected MF and thus putatively assigning compound names to the selected MFs; (iii) MS^1^ and MS^E^ spectra of the selected metabolite candidate and inspection of fragmentation patterns were carefully examined for the structural elucidation on the metabolite; (iv) for the selected metabolite, in-house spectral library and annotation details reported in literature together with a comparative assessment of in silico and experimental fragmentation information were all used for metabolite structural confirmation. Molecular structures with confirmed structural fingerprints were the ones retained.

Pathway analysis of all the annotated and targeted metabolites was done using MetPA (Metabolomics Pathway Analysis), a component of the MetaboAnalyst suite (version 4.0). This was done for the analysis, identification and visualisation of the affected pathways. High-quality KEGG metabolic pathways are used by MetPA as the backend knowledge base. When considering existing literature used, the use of MetPA for pathway analysis in this study provided a framework for the partial mapping of the molecular landscape metabolism understudy which enabled the biological interpretation of the observed changes in the metabolome. For a global visualization of the metabolic changes/alterations, correlation network analysis was done using MetaMapp (http://metamapp.fiehnlab.ucdavis.edu/, accessed on 29 June 2021). This was performed using MetaMapp-encoded chemical structures of the annotated metabolites obtained from KEGG and PubChem databases as well as fold-changes and *p*-values obtained from OPLS-DA-derived descriptive statistics (Table S4). Similarity cut off among metabolites was defined using a Tanimoto score threshold of 0.7, and the generated metabolic networks were visualized using the Cytoscape network visualization software (version 3.8.2) [73].

## 4. Conclusions

Under both well-watered and drought conditions, the application of the microbial-based biostimulant was shown to induce metabolic reconfigurations in both the primary and secondary metabolism. As evident in the targeted and non-targeted approaches, the metabolic readjustments that took place led to the enhanced pool of the selected primary metabolites (i.e., amino acids and the TCA intermediate isocitrate). These can in turn be channelled towards various pathways responsible for the biosynthesis of secondary metabolites such as the phenylpropanoid pathway amongst many others. The majority of the primary and secondary metabolites are involved in plant growth promotion, priming effects as well as stress response, as discussed in this report. In a metabolome view, these differential metabolic patterns and profiles spanned a wide range of metabolic pathways such as phenylalanine metabolism, carbon fixation, alpha-linolenic acid metabolism and the phenylpropanoid pathway related to preconditioning of plant defences and subsequently enhanced drought tolerance and plant growth promotion. The postulated model (from this study) suggested that biochemical and physiological events, induced by the microbial biostimulant in maize plants towards growth promotion include (i) enhanced photorespiration, (ii) improved energy production, (iii) improved root growth for enhanced nutrient and water uptake and (iv) maintained stability of proteins involved in cell division, as well as (v) priming-related mechanisms such as the synthesis of defence-related secondary metabolites and signalling compounds. Furthermore, the drought tolerance mechanisms induced by PGPR application under stress conditions include (i) induced biosynthesis of defence-related metabolites, (ii) enhanced ROS scavenging, (iii) improved osmoprotection, osmotic adjustment and (iv) improved root growth and development for enhanced nutrient uptake. Thus, this study contributes to ongoing efforts to generate knowledge about the molecular and physiological mechanisms underlying biostimulant action. Such fundamental knowledge is essential and a necessary step for advancements and innovations in the biostimulant industry, and for global food security at large.

## Data Availability

The study design information, LC-MS raw data, analyses and data processing information, and the meta-data are being deposited to the EMBL-EBI metabolomics repository–MetaboLights with the identifier MTBLS2955 (https://www.ebi.ac.uk/metabolights/MTBLS2955 accessed on 20 January 2022).

## Supplementary Materials

The following are available online at https://www.mdpi.com/article/10.3390/plants11040510/s1, Figure S1: Chromatographic profiles of maize sap extracts (ESI negative data), Figure S2: Data mining and differential metabolic profiles in maize sap extracts, Table S1: All the putatively annotated metabolites in this study, Table S2: The multiple reaction monitoring MS (MRM-MS) analysis optimal conditions, Table S3: The most impacted metabolic pathways with a pathway impact >0.01, Holm adjusted *p*-value < 0.1 and FDR < 0.1, and Table S4: MetaMapp identifiers.
