# Supplementary material for: Metabolic Circuits in Sap Extracts Reflect the Effects of a Microbial Biostimulant on Maize Metabolism under Drought Conditions
Source: Plants (Basel). 2022 Feb 14;11(4):510. doi: 10.3390/plants11040510 (PMC8877938; doi:10.3390/plants11040510)
Supplement: Supplementary file 1 [file plants-11-00510-s001.zip › plants-1586440-supplementary.pdf]

Supplementary Materials

Section S2.1: Drought stress application

The method described below was used to determine the field capacity (FC) and the permanent wilting point (PWP) of the soil. One-pot containing 17 kg of soil was flooded with water and then sealed on top using a clinging wrap after which, the pot was left for 3 days allowing the water to drain freely from below the pot. The mass of the wet soil was weighed (which represented the FC) following which, the weighed soil was dried in an oven at 50 °C and weighed again, the mass of the dried soil was taken as the PWP. The plant available water (PAW) was then calculated as follows:

(i) PAW = FC – PWP

(ii) 90% PAW = (PAW × 0.9) + PWP

(iii) 50% PAW = (PAW × 0.5) + PWP

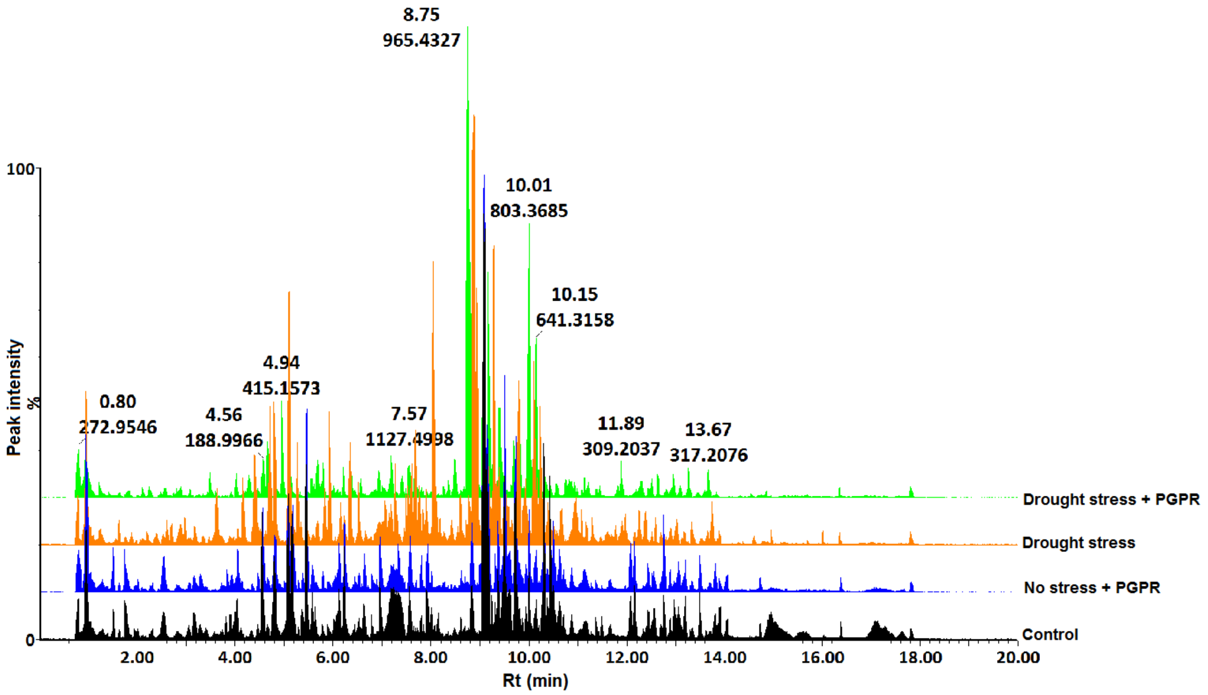

Figure S1: Chromatographic profiles of maize sap extracts (ESI negative data). Representative base peak intensity (BPI) chromatograms comparing the differential peak population of the PGPR-treated to the non-treated control under normal (non-stressed) and drought stress conditions.

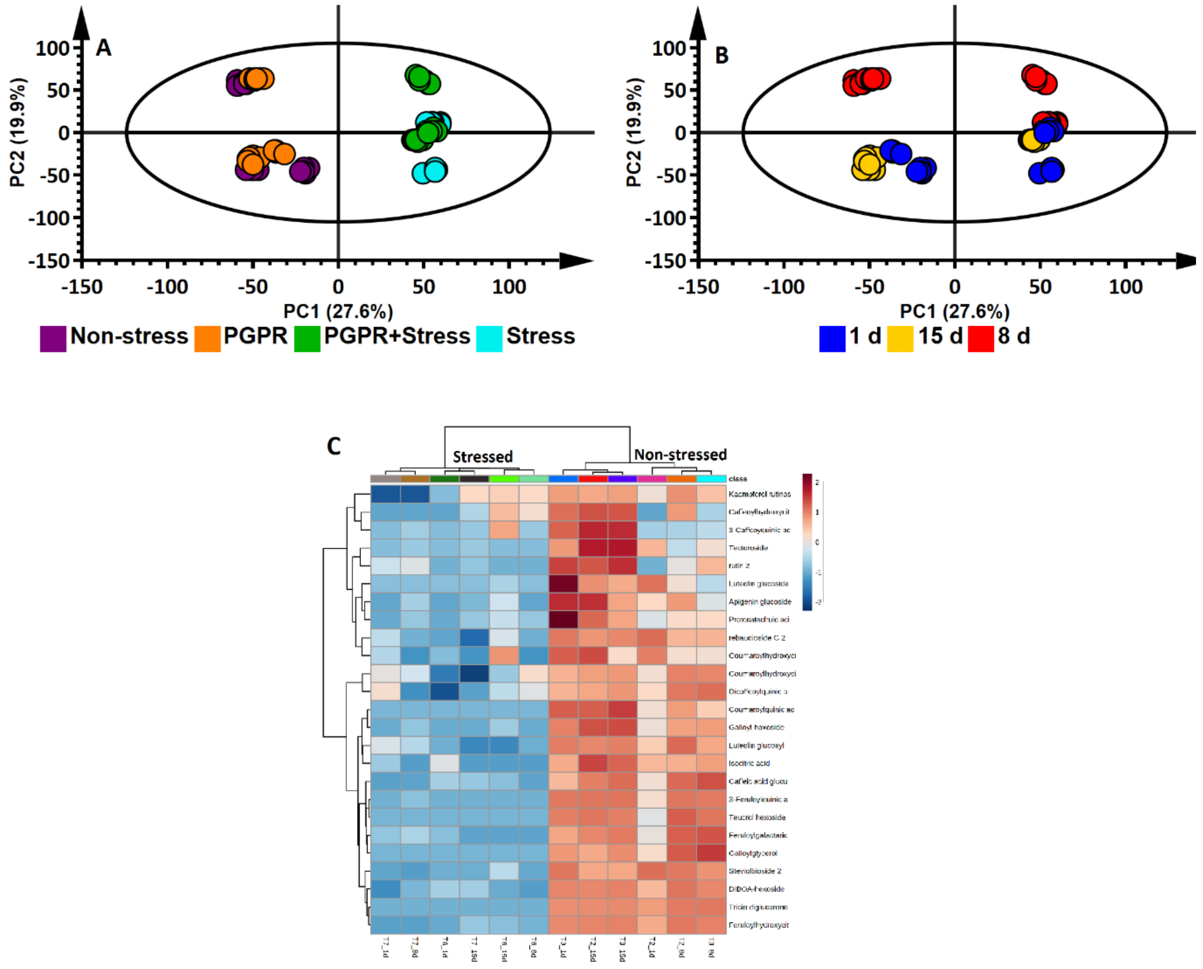

Figure S2: Data mining and differential metabolic profiles in maize sap extracts. (**A and B**) Score plots of an 18-component model that explains 70.3% of the total variation in Pareto-scaled X data with the predictive power of 58.7%, based on seven-fold cross-validation. These PCA score plots show treatment-related sample groupings (**A**) and time-related effects (**B**). **(C**) A heatmap displaying differential quantitative alterations in the levels of metabolites selected based according to PLS-DA VIP evaluation.

Table S1: All the putatively annotated metabolites in this study.

Compound name	Rt (min)	m/z	Fragment ions	Molecular formula	P-VALUE	FC (P/C)
**1-o-galloylglycerol**	1.5	243.057	200,110	$C_{10}H_{12}O_7$	8.2E-06	1.46095
**3-Caffeoyquinic acid**	3.69	353.084	191, 179,135	$C_{16}H_{18}O_9$	0.81893	0.739719
**3-Feruloylquinic acid**	4.56	367.1	193,134	$C_{17}H_{20}O_9$	0.045878	0.889412
**Aconitic Acid**	1.79	173.004	129,85	$C_6H_6O_6$	0.000505	1.23303
**Apigenin glucoside**	4.35	431.151	269	$C_{21}H_{20}O_{10}$	0.287863	0.497961
**Caffeic acid 3-O-glucuronide**	3.3	355.063	209,191	$C_{15}H_{16}O_{10}$	1.13E-06	1.4706
**Caffeoylhydroxycitric acid**	3.95	369.044	207, 189, 127	$C_{15}H_{14}O_{11}$	1.28E-05	0.129162
**Coumaroylhydroxycitric acid**	4.72	353.049	189, 127	$C_{15}H_{14}O_{10}$	0.047433	0.913036
**Coumaroylhydroxycitric acid hexose**	2.64	515.194	353,207		0.316147	0.927524
**Coumaroylquinic acid**	4.09	337.091	163	$C_{16}H_{18}O_8$	0.155289	0.57769
**DIBOA-hexoside**	3.17	342.079	180	$C_{14}H_{17}NO_9$	4.27E-07	0.69784
**Dicaffeoylquinic acid**	3.84	515.122	353,191,179	$C_{25}H_{23}O_{12}$	0.933249	1.03933
**Dicaffeoylquinic acid**	5.13	515.119	353,191,179	$C_{25}H_{23}O_{12}$	0.002877	1.12374
**Diethyl galactarate/succinylglycerol**	1.4	265.088		$C_{10}H_{18}O_8$	0.06124	2.15468
**Dulcoside A/B**	10.43	787.382	641,479	$C_{38}H_{60}O_{17}$	0.135154	0.501296
**Feruloylgalactaric acid**	3.82	385.074	223,209,191	$C_{16}H_{18}O_{11}$	0.001033	1.32471
**Feruloylhydroxycitric acid**	5.09	383.059	189, 127,83	$C_{16}H_{16}O_{11}$	0.008602	0.828243
**Feruloylorientin**	5.33	623.162	431,193,179	$C_{31}H_{28}O_{14}$	0.808106	0.911303
**Galloyl-hexoside (Glucogallin)**	1.88	331.064	169	$C_{13}H_{16}O_{10}$	0.243049	1.08867
**Hydroxy-5-oxo-6,8-octadecadienoic Acid**	12.05	309.203	291	$C_{18}H_{30}O_4$	0.112349	1.0534
**Isocitric acid**	1.27	191.014	173,129,111,85	$C_6H_8O_7$	0.49609	1.30513
**Isovitexin 2''-O-arabinoside**	5.48	563.139	443, 383, 353	$C_{26}H_{28}O_{14}$	2.79E-06	0.808635
**Kaempferol 3-O-rhamnoside-7-O-glucoside**	5.62	593.149	473,429,327	$C_{27}H_{30}O_{15}$	1.34E-06	0.425301
**Kaempferol 3-sophorotrioside**	5.25	771.21	431	$C_{33}H_{40}O_{21}$	0.866256	0.926719
**Kaempferol rutinoside**	7.14	593.151	575, 473,411,337,298,285	$C_{27}H_{28}O_{14}$	9.14E-06	0.595068
**Luteolin glucoside**	6.38	447.092	285	$C_{21}H_{20}O_{11}$	0.11653	0.327733

**Protocatechuic acid hexoside**	3.72	315.068	153	$C_{13}H_{16}O_9$	0.885757	1.1416
**Rebaudioside A/E**	8.93	965.436	803,641	$C_{44}H_{70}O_{23}$	0.001429	1.10858
**rebaudioside C (1)**	9.35	949.44	787,641	$C_{44}H_{70}O_{22}$	0.001651	1.1902
**rebaudioside C (2)**	9.77	949.441	803,787,641,479,473,223	$C_{44}H_{70}O_{22}$	0.492286	1.03732
**rebaudioside D (3)**	8.79	1127.5	965,803,641	$C_{50}H_{80}O_{28}$	2.64E-12	
**Rebaudioside D/I (1)**	7.89	1127.5	803	$C_{50}H_{80}O_{28}$	4.16E-08	1.61184
**rebaudioside F**	9.24	935.424	803,773	$C_{43}H_{68}O_{22}$	0.068323	1.08602
**Rebaudioside I (2)**	8.26	1127.54	965,803,641	$C_{50}H_{80}O_{28}$	0.309541	3.18309
**rebaudioside K/rebaudioside J**	8.72	1111.5	965,787,489,249	$C_{50}H_{80}O_{27}$		
**Rutin**	6.88	609.182	447,431,301	$C_{27}H_{30}O_{16}$	1.9E-05	1.43212
**Steviolbioside 1**	9.99	641.318	479	$C_{32}H_{50}O_{13}$	1E-05	1.46557
**Steviolbioside 2**	10.46	641.317	479,317	$C_{32}H_{50}O_{13}$	1.38E-08	0.477928
**steviolmonoside**	11.35	479.263	317,223	$C_{26}H_{40}O_8$	5.25E-13	0
**Stevioside**	8.98	803.375	641	$C_{38}H_{60}O_{18}$	0.062158	1.08354
**Stevioside 2**	10.09	803.376	641	$C_{38}H_{60}O_{18}$	1.78E-06	0.720085
**Tectoroside**	4.87	587.218		$C_{30}H_{36}O_{12}$	0.018803	3.48567
**Teucrol-O-hexoside**	1.95	477.124	315		3.51E-06	0.721452
**Tricin diglucuronoside**	6.13	681.131	351,329,193	$C_{29}H_{30}O_{19}$	0.013952	1.05845
**Trihydroxyoctadecenoic acid (9,12,13-TriHOME)**	10.75	329.23	293,223,201,171	$C_{18}H_{34}O_5$	0.000605	0

Table S2: The multiple reaction monitoring MS (MRM-MS) analysis optimal conditions.

Compound name	Rt (min)	Ion mode	*m/z*	Transition	CE (eV)	Quadrupole 1 (Q1), V	Quadrupole 3 (Q3), V	Dwell time (msec)
**Amino acids**								
Proline (Pro)	1.503	$[M+H]^+$	116.20	116.20>70.15<br>116.20>43.10	-18.0<br>-28.0	-14.0<br>-13.0	-11.0<br>-15.0	17.0<br>17.0
Cysteine (Cys)	1.317	$[M+H]^+$	241.20	151.90	-14.0	-12.0	-15.0	37.0
Serine (Ser)	1.341	$[M+H]^+$	106.20	106.20>59.95<br>106.20>88.10	-13.0<br>-13.0	-12.0<br>-12.0	-10.0<br>-18.0	17.0<br>17.0
Alanine (Ala)	1.406	$[M+H]^+$	90.20	90.20>44.05<br>90.20>44.90	-13.0<br>-30.0	-10.0<br>-18.0	-15.0<br>-16.0	17.0<br>17.0
Threonine (Thr)	1.409	$[M+H]^+$	120.20	120.20>56.05<br>120.20>74.10	-16.0<br>-12.0	-13.0<br>-13.0	-20.0<br>-28.0	17.0<br>17.0
Aspartic acid (Asp)	1.410	$[M+H]^+$	134.05	134.05>74.10	-15.0	-10.0	-13.0	37.0
Valine (Val)	1.730	$[M+H]^+$	118.20	118.20>72.10<br>118.20> 55.05	-12.0<br>-23.0	-14.0<br>-14.0	-12.0<br>-23.0	17.0<br>17.0
Methionine (Met)	2.142	$[M+H]^+$	150.20	150.20>60.90<br>150.20>56.10	-17.0<br>-24.0	-10.0<br>-11.0	-20.0<br>-24.0	17.0<br>17.0
Tyrosine (Tyr)	3.292	$[M+H]^+$	182.00	182.0>136.10	-14.0	-13.0	-24.0	37.0
Phenylalanine (Phe)	5.929	$[M+H]^+$	166.00	166.00>120.10	-14.0	-12.0	-21.0	131.0
Tryptophan (Trp)	6.774	$[M+H]^+$	205.20	205.20>188.05<br>205.20>146.10	-11.0<br>-17.0	-14.0<br>-14.0	-19.0<br>-14.0	64.0<br>64.0
**Hormones**								
Abscisic acid (ABA)	7.917	$[M+H]^+$	265.10	265.10>247.20<br>265.10>229.30<br>265.10>201.15	-8.0<br>-10.0<br>-13.0	-20.0<br>-13.0<br>-13.0	-20.0<br>-24.0<br>-21.0	65.6<br>65.6<br>65.6
Indole-3-acetic acid (IAA)	26.81	$[M+H]^+$	176.10	176.10>130.10<br>176.10>77.20<br>176.10>103.10	-15.0<br>-43.0<br>-30.0	-20.0<br>-12.0<br>-12.0	-20.0<br>-20.0<br>-22.0	65.6<br>65.6<br>65.6
Zeatin (Zea)	12.984	$[M+H]^+$	220.15	220.15>202.05<br>220.15>136.00<br>220.15>119.00	-19.0<br>-24.0<br>-34.0	-10.0<br>-11.0<br>-10.0	-19.0<br>-24.0<br>-11.0	100.0<br>100.0<br>100.0
Salicylic acid (SA)	22.598	$[M-H]^-$	137.00	137.00>92.95	15.0	20.0	20.0	65.6

				137.00>65.00<br>137.00>75.05	28.0<br>32.0	14.0<br>14.0	10.0<br>27.0	65.6<br>65.6
Amino-cyclopropane carboxylic acid (ACC)	1.58	$[M+H]^+$	101.60	101.60>56.20<br>101.60>28.15<br>101.60>30.20	-14.0<br>-23.0<br>-37.0	-18.0<br>-18.0<br>-18.0	-21.0<br>-10.0<br>-30.0	65.6<br>65.6<br>65.6
Indole-3-carboxaldehyde* (I3A)	16.602	$[M+H]^+$	146.05	146.05	-25.0	-	-	100.0
Indole-3-carboxylic acid* (I3CA)	22.002	$[M+H]^+$	161.95	161.95	-15.0	-	-	100.0

* These compounds did not fragment, thus quantified using single ion monitoring (SIM).

Table S3: The most impacted metabolic pathways with a pathway impact > 0.01, Holm adjusted *P*-value < 0.1 and FDR < 0.1. The **Total** represents all the metabolites in each pathway and the **Hits** indicates the number of matched metabolites from the uploaded data. The **Holm adjusted *p*** is the *p*-value adjusted using the Holm-Bonferroni method and the **FDR** represents the false discovery rates. The **Impact** is the pathway impact from pathway topology analysis.

Indication	Pathway	Total	Hits	Holm adjust	FDR	Impact	Metabolites
	**Significant pathways based on impact factor**						
**1**	Isoquinoline alkaloid biosynthesis	6	1	1	0.5	0.50	Tyr
**2**	Phenylalanine metabolism	11	1	1	0.8	0.47	Phe
**3**	Tryptophan metabolism	28	2	1	0.5	0.32	Trp, IAA
**4**	Glycine, serine and threonine metabolism	33	4	0.3	0.1	0.30	Asp, Ser, Trp, Thr
**5**	Stilbenoid, diarylheptanoid and gingerol biosynthesis	8	2	0.8	0.1	0.26	Asp, Ser, Cys, Met, ACC
**6**	Cysteine and methionine metabolism	46	5	0.1	0.1	0.20	Isocitric acid, Aconitic acid, Ser
**7**	Glyoxylate and dicarboxylate metabolism	29	3	1	0.2	0.15	Chlorogenate (CaQA)
**8**	Alanine, aspartate and glutamate metabolism	22	2	1	0.4	0.13	Asp, Ala
**9**	Aminoacyl-tRNA biosynthesis	46	11	1.03E-08	1.03E-08	0.11	Phe, Cys, Asp, Ser, Met, Val, Ala, Thr, Trp, Tyr, Pro

**10**	Tyrosine metabolism	16	1	1	0.9	0.11	Tyr
**11**	Citrate cycle (TCA cycle)	20	2	1	0.4	0.09	Isocitric acid, Aconitic acid
**12**	Arginine and proline metabolism	34	1	1	1	0.07	Arg, Pro
	Sulfur metabolism	15	2	1	0.3	0.06	Ser, Cys
**13**	Phenylpropanoid biosynthesis	46	3	1	0.4	0.06	Phe, Chlorogenate (CaQA), coumaroylquinic acid
	Flavonoid biosynthesis	47	2	1	0.8	0.05	Chlorogenate (CaQA), coumaroylquinic acid
	Phenylalanine, tyrosine and tryptophan biosynthesis	22	3	0.7	0.1	0.02	Phe, Tyr, Trp
	Glutathione metabolism	26	1	1	1	0.01	Cys
	Carotenoid biosynthesis	43	1	1	1	0.01	ABA
	**Significant pathways based on FDR and P-value**						
**9**	Aminoacyl-tRNA biosynthesis	46	11	1.03E-08	1.03E-08	0.11	Phe, Cys, Asp, Ser, Met, Val, Ala, Thr, Trp, Tyr, Pro
**6**	Cysteine and methionine metabolism	46	5	0.1	0.1	0.20	Isocitric acid, Aconitic acid, Ser
	Cyanoamino acid metabolism	29	4	0.2	0.1	-	
**4**	Glycine, serine and threonine metabolism	33	4	0.3	0.1	0.30	Asp, Ser, Trp, Thr
	Phenylalanine, tyrosine and tryptophan biosynthesis	22	3	0.7	0.1	0.02	Phe, Tyr, Trp

Table S4: MetaMapp identifiers

		Normal conditions (C vs P)		Drought stress conditions (S vs S+P)	
**Compound_Name**	**Abbreviations**	***p*-value**	**Foldchange**	***p*-value**	**Foldchange**
**1-o-galloylglycerol**	Galgly	8,20E-06	1,46	-	-
**3-Caffeoylquinic acid**	3-CafQA	0,82	0,74	0,87	0,81

**3-Feruloylquinic acid**	3-FerQA	0,05	0,89	-	-
**Aconitic Acid**	Ac	5.05E-04	1,23	-	-
**Apigenin glucoside**	Api-glu	0,29	0,50	-	-
**Caffeic acid glucuronide**	Caf-glucu	1,13E-06	1,47	-	-
**Caffeoylhydroxycitric acid**	Caf-hyd	1,28E-05	0,13	0,01	0
**Coumaroylhydroxycitric acid**	Co-hyd	0,05	0,91	0,16	0,47
**Coumaroylquinic acid**	CouQA	0,16	0,58	-	-
**DIBOA-hexoside**	DIBOA-hex	4,27E-07	0,70	0,31	3,77
**1,3-Dicaffeoylquinic acid**	DicafQA1	0,93	1,04	0,07	0,25
**3,4-Dicaffeoylquinic acid**	DicafQA2	2,88E-03	1,12	-	-
**Succinylglycerol**	Suc-gly	0,06	2,15	-	-
**Dulcoside A**	Dul A	0,14	0,50	4,23E-07	1,26
**Feruloylgalactaric acid**	FerGal	1.03E-03	1,32	-	-
**Feruloylhydroxycitric acid**	Fer-hyd	8,60E-03	0,83	-	-
**Feruloylorientin**	FerO	0,82	0,91	-	-
**Glucogallin**	Gluco	0,24	1,09	-	-
**Hydroxy-5-oxo-6,8-octadecadienoic Acid**	H-oct	0,11	1,05	4,07E-11	3,41
**Isocitric acid**	Iso	0,50	1,31	-	-
**Isovitexin 2''-O-arabinoside**	Isovit-Ara	2,79E-06	0,81	0,96	1,01
**Kaempferol 3-O-rhamnoside-7-O-glucoside**	Kae-rha-glu	1,34E-06	0,43	0,13	4,52
**Kaempferol 3-sophorotrioside**	Kae-sop	0,87	0,93	-	-
**Kaempferol rutinoside**	Kae-rut	9,14E-06	0,60	1,56E-12	0
**Luteolin glucoside**	Lut-glu	0,12	0,33	-	-
**Protocatechuic acid glucoside**	Protocat	0,89	1,14	-	-
**Rebaudioside A**	Reb A	1,43E-03	1,11	1,50E-04	1,84
**rebaudioside C (1)**	Reb C	1,65E-03	1,19	0,07	1,16
**Rebaudioside D**	Reb D	4,16E-08	1,61	6,81E-09	0,22
**rebaudioside F**	Reb F	0,07	1,09	0,19	1,09
**Rebaudioside I (2)**	Reb I	0,31	3,18	1,72E-12	0

**rebaudioside J**	Reb J	0,1	0,85	0,01	0
**Rutin**	Rut	1,90E-05	1,43	2,12E-10	34,94
**Steviolbioside (2)**	Steviolbio	1,38E-08	0,48	1,37E-04	1,70
**steviolmonoside**	Steviolmono	5,25E-13	0,1	2,22E-03	1,65
**Stevioside (2)**	Stevioside	1,78E-06	0,72	2,67E-03	1,41
**Tricin diglucuronoside**	Tri-diglucu	0,01	1,06	-	-
**Trihydroxyoctadecenoic acid (9,12,13-TriHOME)**	TriHOME	6,05E-04	0,1	1,09E-04	0,75
**Serine**	Ser	6,80E-18	0,96	6,80E-18	0,74
**Alanine**	Ala	4,19E-19	0,91	4,20E-19	0,62
**Cysteine**	Cys	0,14	1,46	0,01	0,01
**Tryptophan**	Trp	8,14E-21	0,80	8,14E-21	0,26
**Methionine**	Met	4,26E-18	1,08	4,26E-18	0,35
**Tyrosine**	Tyr	1,91E-21	1,04	1,91E-21	0,35
**Phenylalanine**	Phe	3,30E-21	0,89	3,30E-21	0,28
**Aspartic acid**	Asp	3,55E-18	1,55	3,55E-18	0,72
**Threonine**	Thr	4,31E-11	1,13	4,31E-11	0,67
**Valine**	Val	7,75E-21	1,01	7,75E-21	0,34
**Proline**	Pro	1,25E-09	1,62	1,25E-09	0,84
**Indole-3-carboxyaldehyde**	I3A	0,22	0,85	6,81E-08	0,32
**Indole-3-carboxylic acid**	I3CA	0,49	0,84	0,21	1,72
**ABA**	ABA	0,01	4,64	0,41	0,33
**(ACC)1-Aminocyclopropanecarboxylic acid**	ACC	0,03	1,51	1,72E-06	0,13
**salicylic acid (SA)**	SA	3,87E-06	0,86	3,87E-06	0,31
**3-Indoleacetic acid**	IAA	1,4E-04	3,05	0,46	0,69

- Indicates that the particular metabolites were undetected.
